# Rock Surface Strain In Situ Monitoring Affected by Temperature Changes at the Požáry Field Lab (Czechia)

**DOI:** 10.3390/s23042237

**Published:** 2023-02-16

**Authors:** Ondřej Racek, Jan Balek, Marco Loche, Daniel Vích, Jan Blahůt

**Affiliations:** 1 Institute of Rock Structure & Mechanics, Czech Academy of Sciences, V Holešovičkách 41, 182 09 Prague, Czech Republic; 2 Department of Physical Geography and Geoecology, Faculty of Science, Charles University, 128 43 Prague, Czech Republic; 3 Institute of Hydrogeology, Engineering Geology and Applied Geophysics, Charles University, Albertov 6, 128 43 Prague, Czech Republic; 4 Statotest, U Jezu 525/4, 460 01 Liberec, Czech Republic

**Keywords:** monitoring system, strain gauges, rock mass, thermal behavior, slope stability

## Abstract

The evaluation of strain in rock masses is crucial information for slope stability studies. For this purpose, a monitoring system for analyzing surface strain using resistivity strain gauges has been tested. Strain is a function of stress, and it is known that stress affects the mechanical properties of geomaterials and can lead to the destabilization of rock slopes. However, stress is difficult to measure in situ. In industrial practice, resistivity strain gauges are used for strain measurement, allowing even small strain changes to be recorded. This setting of dataloggers is usually expensive and there is no accounting for the influence of exogenous factors. Here, the aim of applying resistivity strain gauges in different configurations to measure surface strain in natural conditions, and to determine how the results are affected by factors such as temperature and incoming solar radiation, has been pursued. Subsequently, these factors were mathematically estimated, and a data processing system was created to process the results of each configuration. Finally, the new strategy was evaluated to measure in situ strain by estimating the effect of temperature. The approach highlighted high theoretical accuracy, hence the ability to detect strain variations in field conditions. Therefore, by adjusting for the influence of temperature, it is potentially possible to measure the deformation trend more accurately, while maintaining a lower cost for the sensors.

## 1. Introduction

The evolution of landscape is strictly connected to endogenous and exogenous processes in natural environments [1,2,3]. Generally, the main factors acting in modeling the landscape are physical and chemical weathering [4,5,6]. In rock media, the stress action is often a consequence of a combination of weathering and atmospheric factors that continuously acts on the rock surface [7,8,9]. In the long term, stress accumulation may affect the mechanical behavior of fractured media and lead to the destabilization of rock slopes. A deep understanding of the main parameters and their uncertainty in affecting this destabilization processes is essential [10]. Among the main factors affecting the stability of rock masses and rock cliffs, the direct effect of temperature [11] and the overall combination of thermo-hydro-mechanical coupling (*THM*) [12,13], has been recognized in the literature. Indeed, temperature can influence the principal parameters in geomaterials, microscopically and macroscopically [14].

Primarily, temperature can affect the stress within the fractures, and drive a change of physical parameters such as permeability as a result of thermal fracturing [15], and/or induce wedging ratcheting [16,17]. The understanding of these comprehensive changes and phenomena is at the base of thermo-mechanical models in geomechanics [13,18], and may allow the prediction of the evolution of the micromorphology at the slope scale.

Unfortunately, in situ data about stresses in rock masses, linked to temperature changes/cycles, are rare and difficult to measure [19]. Usually, to overcome this technical issue, measurements of the strain are used. In industrial or construction practice, resistivity strain gauging is utilized [20], allowing even very small changes in strain to be recorded [21,22]. However, for measurements on the rock surface in natural conditions, strain gauges are influenced by exogenous factors, and the method is practically never used [23]. From this perspective, the requirement to observe the possible influence of external factors on strain measurement, while maintaining lower costs, is evident.

These considerations highlight the importance of proposing a new procedure, dealing with the application of resistivity strain gauging in field conditions. The aim is to determine how the outputs of resistivity strain gauging are influenced by temperature-related factors, discerning the influence on the sensor itself and the rock mass response [24]. Furthermore, the distinction between purely short- and long-term temperature effects is also of interest for understanding the possible influence of global warming [25]. Indeed, climate change can produce an increase in stresses in rock mass fracture networks [26], driving an intensification of instability [27,28], not only in thawing/freezing scenarios [29].

Based on these considerations, a field laboratory test was developed in the new Požáry test site, to monitor the evolution of a controlled natural system. Similar controlled tests can be reproduced at a laboratory scale, simulating natural conditions in a climate chamber [30,31,32]. This instrument allows accounting for the complexity of nature, in which more variables such as humidity, temperature, and thermal cycles act on the evolution of stresses and strain, as shown on small rock samples [30]. Nevertheless, heterogeneous conditions and changes in diurnal temperature cycles detected in situ are difficult to replicate, and a field campaign remains necessary.

On this basis, the objective of the work remains significant, and the applicability of resistivity strain gauging for rock strain dynamics is discussed in detail. Few applications of resistivity strain gauging (in field conditions) are presented in the literature [23,33], and these few do not account for the thermal sensitivity of the sensors. Additional complexity arises from the different configurations of resistivity grids that may influence the results. To account for that, the specific influence of external factors on the outcomes of each strain gauge configuration has been determined, and the use of different strain gauges for specific monitoring tasks is recommended.

In conclusion, assessing the influence of temperature and solar radiation allows us to determine the main causes affecting the development of strain, and possibly predict the consequent rock surface dynamics [1,34]. This study aims to demonstrate the essential importance of accounting for the influence of temperature in field conditionsin the creation of a relatively accessible and easy-to-automate strategy for strain cycle monitoring.

## 2. Materials and Methods

**Figure 1:**
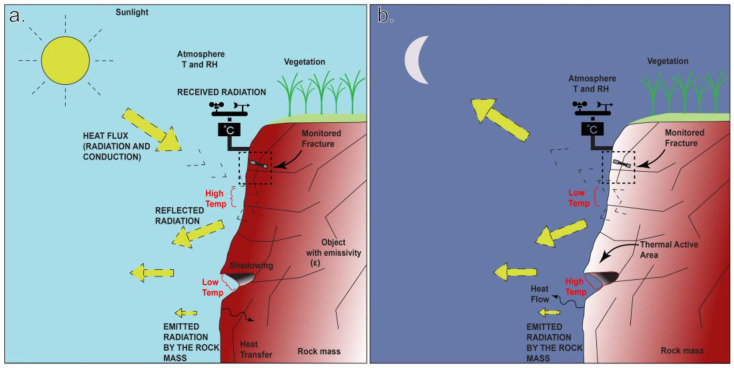
A sketch of the temperature processes in the instrumented fractures at the test site in Požáry (Czechia). (**a**,**b**) Temperature propagates as cyclical input within the active superficial layer, primarily influenced by surface irregularities and exposure. Shadowing and the presence of vegetated areas lead to a possible mismatch between the simulation in the climate chamber and the natural environment, making the test not fully valid for representing the heterogeneity of the natural environment. The dashed rectangle indicates a detail of the instrumented sector of the rock mass. Thermal oscillation experienced by the rock mass in the study area, during the time interval from 16 October to 15 December, ranges from 0 to 18 °C (modified after [35,36,37]).

This section presents the new field laboratory “Požáry test site” and the related installed instrumentation for fractured rock mass deformation detection. A monitoring system, consisting of a weather station, simultaneously detecting superficial and sub-superficial temperature changes, has been implemented, which is able to detect the main thermal processes in the study area (Figure 1). This, along with good accessibility, makes the test site ideal for research. Also in this work, the use of resistivity strain gauges alone is explored in detail.

### 2.1. The Požáry Test Site

**Figure 2:**
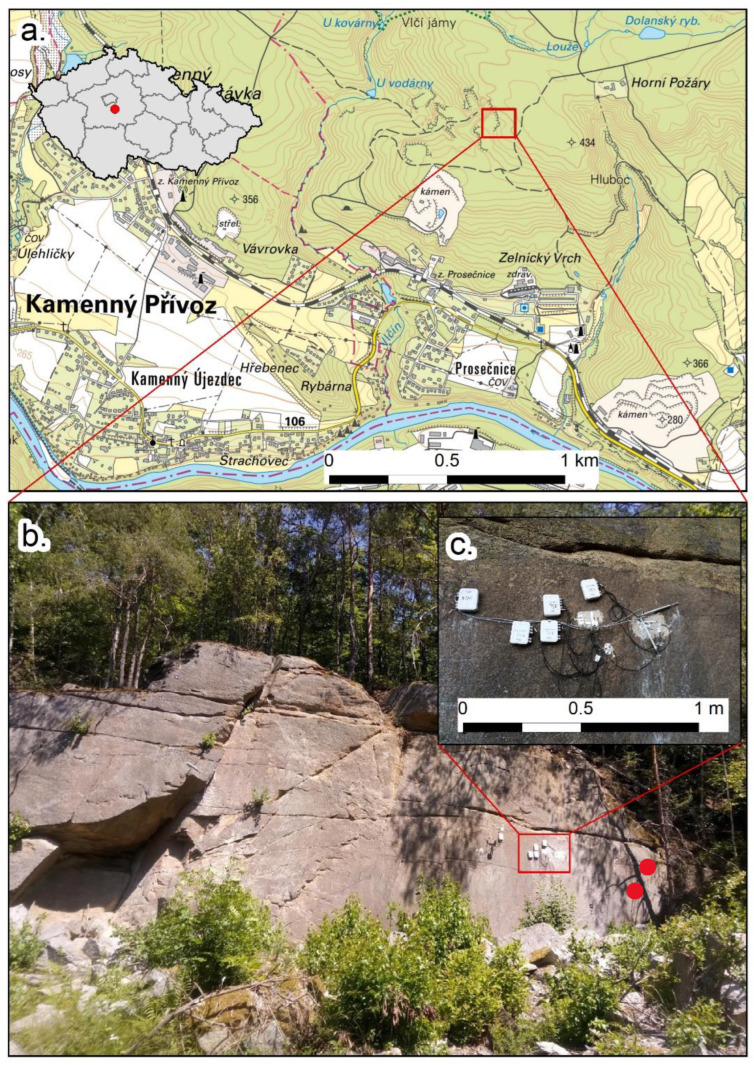
The Požáry test site. (**a**) Location of the field laboratory; (**b**) An overview of the instrumented rock wall; (**c**) Close-up view of the installed strain gauges and induction crack meters. Red dots show the locality where the core samples were collected, representing the rock mass underneath the glued strain gauges.

The Požáry test site is a former pavestone quarry in Central Bohemia (Figure 2a). The quarry was active until the late 1970s, and since then it has developed naturally. Today, the area is made available for scientific purposes, and a network of instruments has been installed, making the test site a fully equipped field laboratory.

Some examples of test sites around the world are described in the literature, analyzing data using numerous approaches and defining various types of destabilizing phenomena [33,38,39]. The novelty of our study area lies in the different latitude of the location, in which, from a climatic perspective, it is possible to observe a different range of temperatures, compared with the other test sites worldwide. These thermal fluctuations potentially affect the volumetric expansion of fractures and drive an irreversible evolution, often after intense and prolonged cycles of alternating extreme hot and cold temperatures, in different ways, based on geographical area [7,38,40]. In fact, distinct locations present incomparable temperature series characterized by diverse amplitudes.

From a geomorphological point of view, the rock wall is relatively flat (average dip angle close to 90°), with a west aspect (W). The rock type is classified as biotitic granodiorite or quartz diorite [41,42]. The area is part of the Central Bohemian Plutonic Complex [43], and the Požáry intrusion forms an extensive body, which was radiometrically dated 351 ± 11 Ma [44]. The whole outcrop was disturbed in the past, both tectonically and due to blasting, which led to horizontal rock mass alterations (Figure 2b). For these reasons, in situ conditions are relatively complicated, and the properties of the rock mass differ even within relatively small distances. Table 1 shows how the variation of some parameters such as Young Modulus (E_d_) and Bulk Modulus (Kd) change considerably in the examined samples, collected near the instrumented area.

Figure 2b,c show the rock wall where the monitoring system, which was proven functional at other sites [45], was established. On the rock mass was installed a series of eleven strain gauges (with different configurations explained and presented in Table 2 and Table 3), for the purpose of monitoring the possible plastic strain over the microfracture in a short period of two months. Moreover, in the absence of plastic response, the data may be useful for determining the short-term elastic behavior of both intact rock and microfractures. The data were collected from the Požáry test site through an IoT network database and then analyzed. This system allows continuous monitoring of meteorological changes (together with the solar radiation balance), and an in-depth temperature profile up to 3 m [45]. Surface and joint dynamics were monitored using a set of electrical resistivity strain gauges (for current testing) and conventional induction crack meters.

### 2.2. Resistivity Strain Gauges

Resistivity strain gauging is a well-known method of indirect strain measurement, applied since the 1960s [46]. Strain gauge sensors can detect surface strain variations using the principle of measuring changes in the resistivity of a conductive grid or wire [47]. By applying strain to the grid, the wire cross-section reduces, which results in a decline in conductivity. This simple principle makes strain gauges cheap to manufacture and very effective in measuring even small surface strain changes. The surface strain is transmitted through the adhesive to the foil, with the conductive wire, which changes its resistivity. This can be expressed using the gauge factor (*GF*), as shown in Equation (1).

(1) $$GF=\frac{\Delta R/R}{\varepsilon }$$

**Figure A1:**
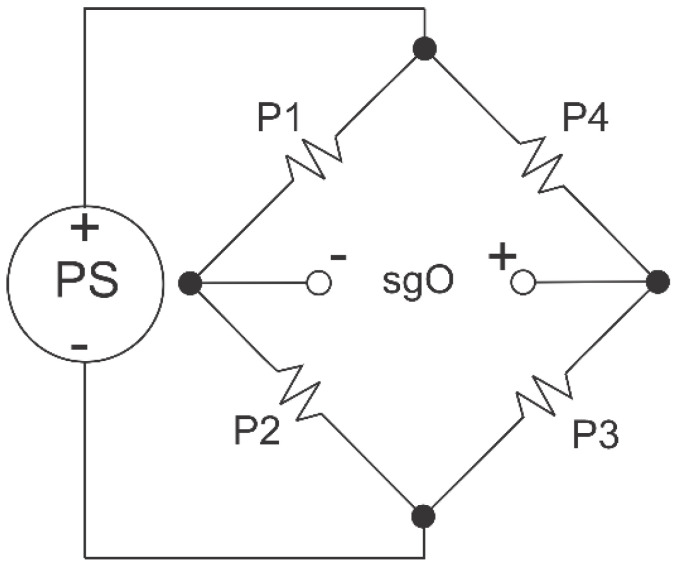
*Wheatstone bridge* scheme. P1–4: positions of gauges/resistors, PS: power source, sgO: strain gauges output. Strain gauges are configured in Wheatstone bridge circuits to detect small changes in resistance.

*GF* describes how the resistivity (*R*) of the strain gauge changes in proportion to applied strain (*ε*), and can be associated with the sensitivity coefficient of the strain gauge. Usual metal foil in strain gauges presents a common value of *GF* ≈ 2 [48]. Moreover, strain gauges can differ by resistivity, dimensions, and layout. Resistivity can have an influence on power requirement and thermal stability [46], while the dimensions and layout may be different for different applications [49]. The strain gauges are wired using the *Wheatstone bridge* [50,51], which allows the detection of exceedingly small resistivity changes. The *Wheatstone bridge’s* four positions can be occupied by active strain gauges, dummy strain gauges, or resistors. By a combination of these components and their reciprocal orientation, several configurations can be wired [52]. The possible configurations, and their different utilities are explained in Table 2, which describes the most common ways to wire the *Wheatstone bridge* (See Figure A1 in Appendix A).

Resistivity strain gauges are used extensively in the engineering field for strain measurements of homogeneous materials, such as metals or plastics, in controlled laboratory conditions [53]. In the geosciences, strain gauges are used in rock sample laboratory testing to record strain evolution during compression or tension tests [20,24,54]. Their relative affordability makes these perfect for laboratory use, especially for strain measurement during uniaxial testing [55]. A further field of application lies within civil engineering [56,57], in which these devices are used to measure strain within a concrete, rebar, or steel construction, and for monitoring during tunneling [58] and mining activities [59]. Specialized probes were also developed based on strain gauges, which are able to measure stress changes in the rock mass depth, using state-of-the-art, but financially demanding, dataloggers [60,61]. For these rock mass depth applications, the temperature is considered stable; consequently, the instruments are not compensated for possible thermal effects [62]. Generalizing, the application of resistivity strain gauging under natural rock slope conditions has not been practically investigated. This is surprising, given the capacity of thermal forces to continuously act on fractured rock masses, inducing irreversible/plastic deformations. The cumulative stress concentration along fractures induces and promotes irreversible strains [13,63], often related to cyclical thermal fatigue [40,64], which is responsible for a decrease in rock stability [65].

The instruments can be used for both long- and short-term applications [23,33], but a meaningful change in the long-term resistivity of the wires may be caused by thermo-cyclic loading (long term drifting), and it can cause inexplicable changes in the rock strain trends [66,67].

Additional complexity arises from the choice of the right configuration. It is well known that the grid length should be based on the type of expected results [68]. More heterogenous material may permit the use of longer strain gauges, for capturing the general (average) rock mass deformation, while short grids may detect the mineralogical thermal expansion of partial grains. The smaller the scale of the investigation, the shorter the length of the grid should be. In our study, two grid lengths were adopted, aiming at understanding the different behavior of short and long strain gauges in the studied material (granodiorite).

To determine firstly the capability of the method in natural conditions, secondly the different shapes’ response, and thirdly how the instrument’s results are influenced by temperature cycles, strain gauges were tested in different configurations and types, listed in Table 3. These settings of strain gauges on intact rock and over the microcracks were compared, and the data were collected through the IoT network database.

**Figure 3:**
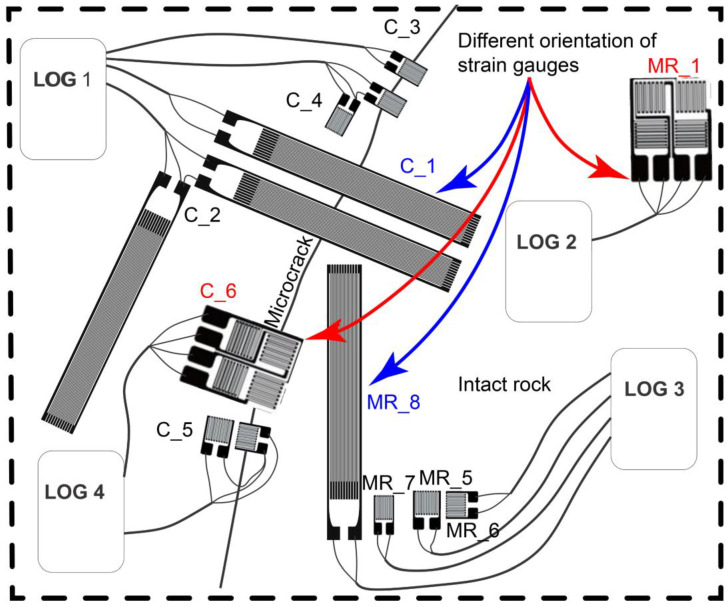
Sketch of strain gauge configurations. The figure shows the location of the instruments and the detection of both intact rock and microcrack. The configurations of instruments are presented with their different shapes and orientations (red and blue color) for testing possible distinct behavior under temperature changes.

Practically, strain gauges were installed on the rock face, as shown in Figure 2c and Figure 3, and the different sensor outcomes, based on several configurations, were compared. The results of this comparison are presented in the following section (See Section 3).

### 2.3. Data Processing

**Figure 4:**
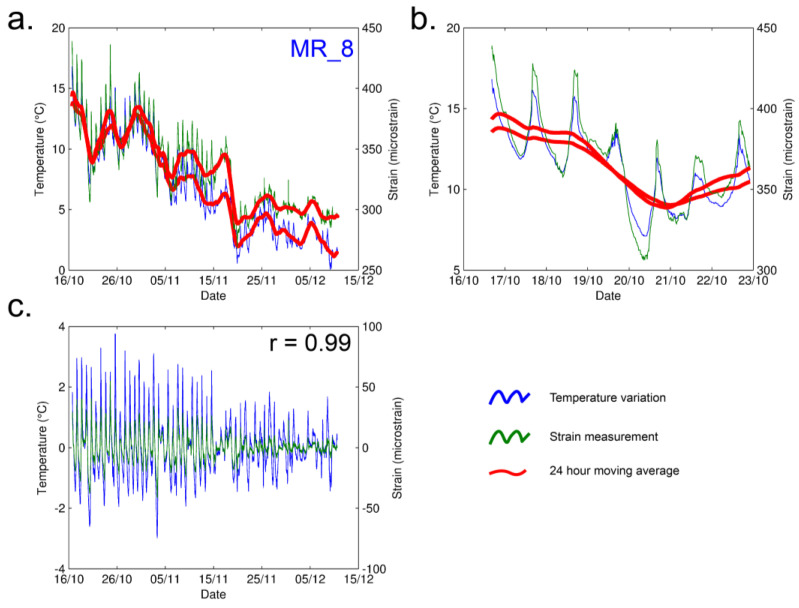
Data processing. (**a**) Evaluation of measured temperature variation in blue, strain measurements using ¼ bridge (code MR_8) in green, and 24 h moving average in red. MR_8 was placed in the intact rock and was used as a reference for the behavior of the massive rock; (**b**,**c**) Example of daily variation of temperature (blue) and strain (green), in which Pearson correlation coefficient of 0.99 indicates a very high correlation between these two variables, computed using Equation (3).

**Figure A2:**
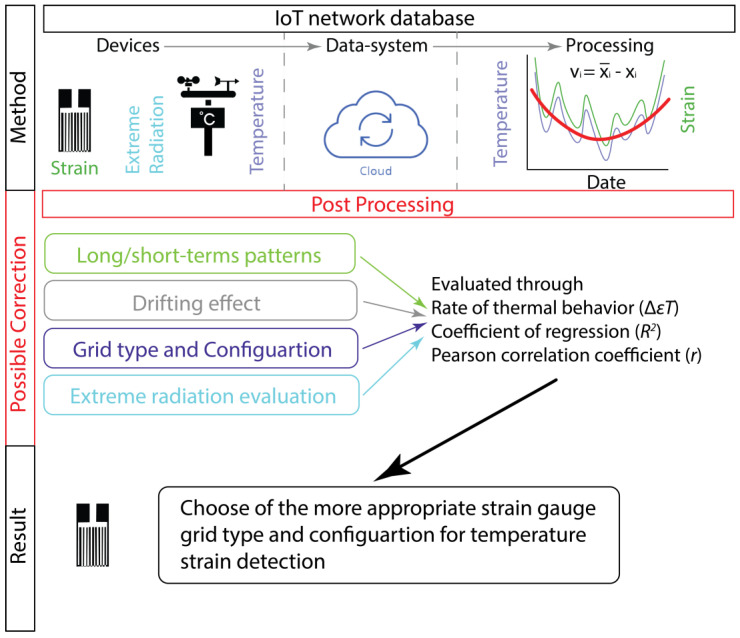
Methodological flow chart. An IoT network allowed to transmit and process the data from the installed devices in situ. In a post processing stage, an accurate estimation of the main factors affecting the sensors are evaluated through mathematical formulations. Finally, a result highlighting the feasibility of strain gauges for our specific case study is presented.

As previously mentioned, the application of the method required an intensive post processing stage (Figure A2). Time series of temperature and solar radiation were considered, to exclude possible interaction with the strain measurements (Figure 4), during the autumn season (from 16 October to 15 December). The temperature was acquired through a weather station (Figure 2b).

Changes in temperature may affect sensors, and when high accuracy is required, it is necessary to calibrate and compensate for thermally induced resistivity changes. In the case of strain gauges, there is the possibility of hardware compensation for the thermal behavior on the measured strain using half and/or full bridges. However, a primary task should be to verify possible relationships and to quantify the dependencies between the variables in the various configurations. This was done using linear regression and simple correlations, aiming at describing the possible influence of temperature on each strain gauge configuration presented in Table 3. To do so, the Pearson correlation coefficient (*r*) was utilized to identify patterns in the data, while the coefficient of determination (*R*²) was used to identify the strength of such dependencies. The use of the Pearson correlation coefficient, given the linear relationship and normal distribution of the variables, was hypothesized, and, once established, the pattern and strength a filter step was proposed. Furthermore, the resulting measurements of strain were considered for a post processing stage.

Considering the time series in Figure 4a,b, intermediate-term (monthly) and short-term (daily) temperature variations were discerned. It is possible to observe a constant daily period in intervals of 24 h (Figure 4b) and assume that the drifting effect of the measured strain for the short period of measurement is negligible.

**Figure 5:**
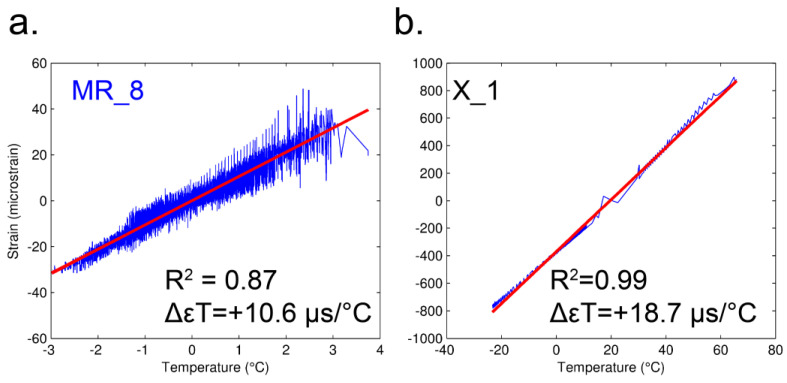
(**a**) Rate of the thermal behavior of measured strain (blue) quantified using linear regression (red line) for MR_8 in situ; (**b**) Rate of thermal behavior of measured strain (blue) quantified using linear regression (red line) for X_1 in climatic chamber. MR_8, C_1 and X_1 have the same configuration of sensors, but all three are different and distinct pieces.

Theoretically, the final strain may be affected by the temperature at several levels. For instance, the resistance of the strain gauge wire changes with temperature changes. Furthermore, temperature influences the size of the strain gauge foil because of the thermal expansion of its metal. Finally, actual dimensional change of the measured rock material, which is caused by natural processes and/or thermal expansion of the rock mass itself, can be estimated. These effects work together and cannot be easily separated. To account for this, the relationship between the temperature and strain series was defined and quantified. For this purpose, the daily variations of temperature with the daily variations of the measured strain were compared, both in the field and in the climate chamber. The daily variations ($v_{i}$) were evaluated using Equation (2):

(2) $$v_{i}=\bar{x_{i}}-x_{i},$$

where $v_{i}$ is computed as the deviations of measured value ($x_{i}$) from the 24 h moving average ($\bar{x_{i}}$). The relationship between the temperature and strain measurements was estimated using the Pearson correlation coefficient. Furthermore, to obtain the exact rate of the correlation, a simple linear regression on the sorted residuals was computed (Figure 5). The linear regressions show a dependency between the two variables and allows us to state that the relationship is valid in both the daily variations (deviations from daily mean) observed in situ (−3 + 4 °C) and simulated in the climate chamber (−20 + 70 °C).

## 3. Results and Discussion

We calculated the rate of thermal behavior (Δ*εT*), coefficient of determination (*R*²), and Pearson correlation coefficient (*r*) of each strain gauge and for a reference foil in the climate chamber (Table 4). The results of these preliminary tests demonstrated the high mutual dependency between the temperature and strain series. The majority of strain gauges showed a correlation in a range of R^2^ = 0.8–0.99, while the Pearson correlation coefficient was around *r* = 0.91–0.99. However, we identified three strain gauges (C_2, C_4 and C_5), which were not fully correlated with temperature signals (R^2^ = 0.1–0.5 and *r* = 0.3–0.71). These strain gauges belong to a configuration T1 ½ bridge with a variable grid type of 100 mm/120 Ω, 10 mm/120 Ω, and 6 mm/350 Ω. The results are partially in accordance with the literature, in which the T1 ½ bridge should provide automatic analogue temperature compensation (Table 2).

Figure 5 shows the correlation of the coupled thermal effect on both rock material and strain gauge sensor (foil). To separate these two effects, and detect only the deformation of the rock mass, we recorded time deformation series for the unbonded strain gauge (code X_1). Measuring the behavior of the sensor, artificially simulating diurnal cycles within a climatic chamber, permits hypothesizing the filtration of the results of the strain gauges in a post processing stage. The rate of the temperature dependence on the foil (code X_1) was mathematically estimated (in the same way as for the field) and values of Δ*εT* = 18.9 µs/°C, R^2^ = 0.99, and *p* = 0.99 were returned, as shown in Table 4 and Figure 5b. The results clearly highlight the linear dependency of the behavior of the foil under temperature changes.

Overall, mathematical analyses have already confirmed the relationship between temperature and strain in the majority of sensors (Table 4), mostly in ¼ bridges. This phase revealed its importance in practice, and that it should be considered in all possible monitoring system applications, to properly define the dependency of the sensors on temperature. In addition, for short term campaigns, the strain gauges should be constructed to compensate for the temperature-related behavior of specific materials [62].

**Figure 6:**
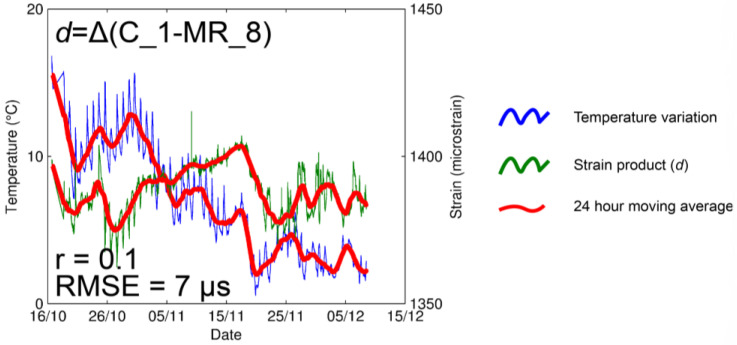
Comparison between strain gauges C_1 and MR_8. The blue line shows the time series of temperature, while the green line represents the product of the difference between C_1 and MR_8 time series of the strain (*d*) computed using Equation (3). Pearson correlation coefficient demonstrates no dependency between the value of *d* and the temperature series. In addition, the time series shows no significant trend or predicted error (*RMSE*).

We then proceeded to consider the possible strain gauge drifting effect, in the selected monitoring period. To evaluate for this tendency of the instruments, we decided to use a pair of identical strain gauges (grid type 100 mm/120 Ω). The first gauge (code MR_8), used as a reference, was placed on an intact rock (ε), while the second (code C_1) was placed over a microcrack (*ε_R_*) at a distance of a few centimeters apart (Figure 3). We highlight here that the two instruments have the same configuration, based on 100 mm/120 Ω and T1 ¼ bridge. Hence, we assumed that both gauges were affected by temperature and drift in the same manner, therefore, gauge degradation and configuration were probabilistically identical. Figure 6, shows the results, pointing out an analogous thermal behavior for both strain gauges. The computed difference between the two devices (*d*) was calculated using the following Equation (3):

(3) $$d=\varepsilon -\varepsilon_{R}$$

It shows no significant intermediate-term trend or significant temperature dependence, and the Pearson correlation coefficient for those discrete functions confirmed a very low value (*r* = 0.10). In addition, the dispersion of differential time series was described by Root-Mean-Square Error with *RMSE* = 7 µs. Based on the comparison performed, strain gauges with identical parameters, C_1 and MR_8 with length 100 mm and resistivity 120 Ω and configuration T1 ¼ bridge, from the same series, demonstrated remarkably comparable properties. This is reflected in the similar reaction to temperature changes and similar trends, even though they were placed respectively over a microcrack and on intact rock. Notably, given these results, we can assume that the measured microcrack remained inactive during the testing (autumn season). This can be caused by small temperature oscillations in the investigated time period, that may different in a different season.

**Figure 7:**
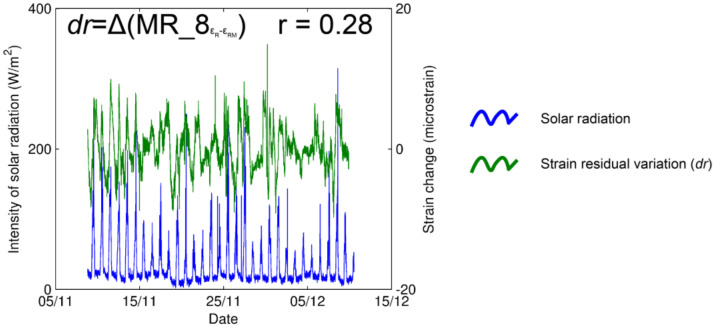
Strain residual variations (*dr*) are not statistically explained by temperature influence (green) or solar radiation (blue) in the strain gauge MR_8. This analysis was performed for the strain gauge placed in intact rock; sensors over the microcrack may still be potentially influenced by extreme radiation.

Moreover, it was assumed that the output of the sensors could be possibly affected by extreme solar radiation, which can cause uneven warming of the device. This can be produced by a lack of insulation in the reflective aluminum foil that covers the devices. Since solar radiation constituted a daily period, we could compare diurnal variations of the strain to modelled variations based on the estimated values of thermal behavior (Δ*εT*, reported in Table 4), to assess a value of dependency. Figure 7 shows how the residuals of strain measurements cannot be explained by the peaks in solar radiation in the case of the strain gauge MR_8. In other words, we selected the maximum values of solar radiation and expected to observe a response in the strain measurements in the intact rock (anomalous increase of deformation). To do so, we subtracted the daily measured strain variations ($\varepsilon_{R}$) from the modelled daily strain variations ($\varepsilon_{RM}$), which resulted in a series of values which are not explained by the temperature influence, so-called residuals (*dr*), computed following the Equation (4):

(4) $$dr=\varepsilon_{R}-\varepsilon_{RM},$$

where

(5) $$\varepsilon_{RM}=v_{T}\;\;.\;\;du_{T}\;$$

and $v_{T}\;$is in accordance with Equation (2). As a result, Figure 7 does not show, visually or statistically (*r* = 0.28), a dependency between the variables, permitting us to presume no effect of solar radiation on the measurements and/or sensors. From this result, in our case, the strain gauges were not affected by direct solar radiation on the intact rock surface.

Additionally, the grid type (ratio between length and resistivity) and configuration of strain gauges are valuable information to consider. The same grid type presented the same order of rate of thermal behavior (e.g., Δ*εT* = 10.6–12.6), as in the case of 100 mm/120 Ω for configuration T1 ¼ bridge (C_1 and MR_8). Interestingly, decreasing the value of the grid type, we noticed a proportional increase in the value of their associated thermal rate behavior (Table 4), suggesting that a sensor with high resistivity in a short distance is able to detect higher thermal activities. This suggests that specifying the scale of the study and a particular length and configuration of strain gauges is a good practice. In fact, a given length of strain gauge cannot be generally valid for all materials. We stress again that these results may vary based on the mineralogical content of rocks, and outputs should be evaluated case by case. Furthermore, the comparison of different configurations, in this perspective, is quite complicated to perform due to the different lithological conditions, and should be systematically studied.

We observed that the measurements of variations of strain in situ are affected by a combination of temperature dependence of the sensors and thermal expansion of the rock. Indeed, microcracks proved to be inactive in the monitored period, and we assumed that thermal expansion may be the exclusive physical mechanism detectable by the instruments. As previously mentioned, we tried to estimate the temperature dependence of the ¼ bridge sensor itself by doing a laboratory experiment in the climate chamber. Comparing the sensor’s response to temperature (code X_1, Table 4) and the response estimated from the field data (code MR_8, Table 4), we attempted to extract the actual deformation of the measured granodiorite. The calculated values of the strain, which we obtained, are far from corresponding to the tabulated values of the thermal expansion of the measured material (α = 7.7 × 10^−6^ per degree Celsius [65,69]). This is probably caused by an erroneous response of the freely placed foil sensor in the climate chamber, which cannot be compared to a foil fixed to a material of different thermal expansion, and it is highly affected by the placement surface and glue material. Although, for the sensor with configuration 100 mm/120 Ω (code C_1), we hypothesized to filter the thermal effect on the foil (that we tested in the climate chamber), the results of the subtraction of these two values gave a result that did not correspond to the real thermal expansion of granodiorite. Recall that we did not estimate plastic deformations nor any substantial difference between microcracks and intact rock. This suggests that the only possible process and value detectable from the instrument is the thermal expansion of the material. This notwithstanding, sensors with configuration 100 mm/120 Ω and T1 ¼ bridge have shown reasonable strain values, without a filtering stage, in accordance with α.

## 4. Conclusions

The goal of this test, in the new Požáry test site, was to evaluate the use of strain gauges and to determine how the devices are affected by temperature. The adaptation of a well-known industrial protocol was applied to a complex natural condition, for testing the capability to record thermally induced plastic and elastic deformations in a reasonable time window (monthly). The novelty of the method was applied systematically for measuring deformations on the surface of rock outcrops, to help detect elastic strain and potentially observe plastic deformations, which, in the context of rock fatigue and accumulation cycles, is critical to monitor in instable fractures. In this work, we carried out field tests of various strain gauges, wired in different arrangements, on the intact rock surface and on microcracks.

To account for the complexity in determining the influence of thermal effect on the outputs of individual strain gauges, we evaluated three grid configurations (full bridge, half bridge and quarter bridge).

Interestingly, in our case, the same strain gauges on massive rock and microcrack show similar thermally induced behavior, suggesting that there were no spatial changes on the microcracks and intact rock during the monitoring period (2 months). The microcracks may not have been active within the temperature interval investigated, or are perhaps not active at all. Note that all measurements were performed during positive temperatures, and it is possible to expect different behavior whether temperature amplitudes (freezing/thawing) are larger and more frequent. On the other hand, short-term temperature variations with a period of 24 h have a strong influence on measurements of ¼ bridge strain gauges (Δ*εT* = 5.1–12.6 µs/°C), while time series of ½ bridges also exhibit periodic short term variations (Δ*εT* = 0.4–1.9 µs/°C), albeit with significantly smaller amplitudes (Table 4), confirming the partial temperature compensation.

Moreover, the relationship between extreme radiation and strain measurements in the computed residual peaks demonstrated no dependency (Figure 7). This proves that aluminum covers can adequately protect the strain gauge foils from solar-radiation-induced heating.

In conclusion, the results of the work clearly point out the difficulties of using strain gauges for in situ rock mass deformation detection. This can explain why, until now, only a few studies have used the technique directly on the outcrop surface. The location and durability of the dataloggers still remain to be carefully evaluated, together with the wires, which should be kept as short as possible to reduce the temperature effects. This should be performed by the self-compensating layout of devices, or by advanced data processing methods. In fact, in the case of our data, most of the strain variation can be explained by temperature and cannot be decoupled. Considering all the results of the different strain gauges configurations, we observed that the variations of strain that we monitored are affected by the external temperature. This makes the sensors useful for the determination of the specific strains induced by short-term (diurnal) thermal effects in the configuration of quarter bridges, while discarding the use of half-bridges, which were able to compensate for the temperature effect. Specifically, strain gauges C_4 and C_5 were not statistically affected by the temperature. Based on our observations, the method is not yet suitable for measuring relative strain changes over the time of two months, and longer-term cycles are, in fact, harder to observe because of possible long-term drift. This is not always valid and some examples have proven the use of the system in long term monitoring [33]. This can be due to the different manufacture of strain gauges, different configuration, or different rock response, but the feasibility still remains to be analyzed case by case.

Longer gauges should be used to measure rock mass strain, while shorter ones should be used to measure the behavior of the specific rock slope elements, such as microcracks or partial mineral grains. Strain gauges should be able to measure non-thermally linked activity when thermally induced strain changes are easily separable by further data processing or different configuration settings. However, we were not able to systematically decouple the effect of temperature from the sensor’s thermal influence, and the results still remain within a threshold of uncertainty.

On the basis of these considerations, we can suggest the use of a quarter bridge (simplest configuration) for short-term strain cycle evaluation, once the thermal effect on a specific strain gauge is known, and in situ measurements can be used to measure the thermal expansion of specific rock types. These data are crucial inputs for all rock-mass-related numerical models, and field data should represent the real state of rock slope more accurately than the values investigated from small rock mass samples. We emphasize again that, to the authors’ knowledge, the use of strain gauges has not been systematically used in natural conditions, and for this reason, and the low budget required for installation, they are an important resource. However, we encourage the careful use of strain gauges to fit the scale of the experiments. The resistivity strain gauging method showed potential for in situ strain measurement, but failed in measuring, systematically, realistic values of deformation in all the configurations. This is an opportunity for future work, which will follow. Finally, the method can be used for measuring the relative temperature-caused strain or for non-thermally linked strain irregularities such as crack openings. Furthermore, the selected field laboratory site at Požáry made it possible to fully understand the interaction of natural processes, due to the use of a monitoring system in a considerable time window, and through new instrumentation, contributing a step forward in the understanding of physical processes acting within the rock slope.

## Figures and Tables

**Table 1:** Physical and mechanical properties of ten rock samples from the tested area. Primary wave (Vp), secondary waves (Vs), rock density (ρ), Young Modulus (Ed), shear modulus (μd), Poisson’s Ratio (νd) and bulk modulus (Kd) were measured and calculated. Samples were collected at the base of the outcrop near the instrumented area.

Parameters	V_P_	V_S_	ρ	E_d_	μ_d_	ν_d_	K_d_
Units	[km/s]	[km/s]	[g/cm^3^]	[GPa]	[Gpa]	[-]	[GPa]
Mean	2.599	1.604	2.614	16.191	6.832	0.187	8.814
STD	0.316	0.198	0.019	4.580	1.969	0.052	2.599

**Table 2:** Different type of configurations for strain gauges and short descriptions of their utilities.

Type	Geometric Pattern	Description
Quarter bridge T 1	One active gauge and three dummy resistors.	This configuration is used for uniaxial strain measurements and is not temperature-compensated.
Quarter bridge T 2	One active gauge, one passive transverse to applied strain mounted gauge, and two dummy resistors.	This configuration is temperature-compensated by the dummy gauge, which is only affected by temperature and not by the applied strain, because it is unbonded to the material. This configuration is frequently mistaken for half bridge type 1.
Half bridge T 1	Two perpendicularly bonded gauges and two resistors.	One gauge mounted in the expected direction of the strain, while the second is placed perpendicularly. The secondary gauge bonded on the same material provides temperature compensation.
Half bridge T 2	Two parallel bonded gauges and two resistors.	This configuration is used in the case of measuring bending strain. It measures both compression and tension on opposite sides of the tested beam. It is temperature-compensated, but it is not suitable for surface strain measurements.
Full bridge T 1	Four active gauges.	Two opposite-facing diagonal pairs, ideal for a bending strain measurement. This configuration provides temperature compensation.
Full bridge T 2	Four active gauges.	One diagonal pair and a second perpendicular pair of gauges. Bending strain application. This configuration provides temperature compensation.
Full bridge T 3	Four active gauges.	Two perpendicularly facing gauges for compressive and tensile strain measurements in two axes.

**Table 3:** Strain gauge types and their configurations used in the study area.

Code	Placement	Grid Type	Configuration
C_1	microcrack	100 mm/120 Ω	T1 ¼ bridge
C_2	microcrack	100 mm/120 Ω	T1 ½ bridge
C_3	microcrack	10 mm/120 Ω	T1 ¼ bridge
C_4	microcrack	10 mm/120 Ω	T1 ½ bridge
C_5	microcrack	6 mm/350 Ω	T1 ½ bridge
C_6	microcrack	6 mm/120 Ω	T3 1/1 bridge
MR_1	Intact rock	6 mm/120 Ω	T3 1/1 bridge
MR_5	Intact rock	6 mm/350 Ω	T1 ¼ bridge
MR_6	Intact rock	6 mm/350 Ω	T1 ¼ bridge
MR_7	Intact rock	10 mm/120 Ω	T1 ¼ bridge
MR_8	Intact rock	100 mm/120 Ω	T1 ¼ bridge
X_1	Climate chamber	100 mm/120 Ω	T1 ¼ bridge

**Table 4:** Results for strain gauge codes and their rate of thermal behavior (Δ*εT*), coefficient of regression (*R^2^*), and Pearson correlation coefficient (*r*). In red, configurations that are less affected by temperature and should be compensated by definition.

Code	Rate of Thermal Behavior (Δ*εT*) [µs/°C]	Coefficient of Regression (*R*^2^) [-]	Pearson Correlation Coefficient (*r*) [-]
C_1	12.6	0.99	0.99
C_2	1.9	0.50	0.71
C_3	5.1	0.83	0.91
C_4	0.4	0.44	0.66
C_5	0.4	0.10	0.30
C_6	5.4	0.89	0.94
MR_1	13.4	0.98	0.98
MR_5	49.1	0.96	0.98
MR_6	52.4	0.95	0.98
MR_7	16.1	0.95	0.97
MR_8	10.6	0.87	0.93
X_1	18.7	0.99	0.99

## Data Availability

Elaborated data are presented in the manuscript. Raw experimental data can be provided by the authors upon reasonable request.

## Appendix A

To measure small changes in resistance, strain gauge configurations are based on the concept of a *Wheatstone bridge*, illustrated in Figure A1, in which a network of four resistive arms, with an excitation voltage, is applied across the bridge. The *Wheatstone bridge* is the electrical equivalent of two parallel voltage divider circuits. P1 and P2 compose one voltage divider circuit, and P4 and P3 compose the second voltage divider circuit. The output of a *Wheatstone bridge* is measured between the center nodes of the two voltage dividers.
